# Age- and time-of-day dependence of glymphatic function in the human brain measured via two diffusion MRI methods

**DOI:** 10.3389/fnagi.2023.1173221

**Published:** 2023-05-22

**Authors:** Guangxu Han, Ying Zhou, Kemeng Zhang, Bingjie Jiao, Junwen Hu, Yifan Zhang, Zejun Wang, Min Lou, Ruiliang Bai

**Affiliations:** ^1^Department of Physical Medicine and Rehabilitation of the Affiliated Sir Run Shaw Hospital AND Interdisciplinary Institute of Neuroscience and Technology, School of Medicine, Zhejiang University, Hangzhou, China; ^2^Key Laboratory of Biomedical Engineering of Education Ministry, College of Biomedical Engineering and Instrument Science, Zhejiang University, Hangzhou, China; ^3^Department of Neurology, School of Medicine, The Second Affiliated Hospital of Zhejiang University, Hangzhou, China; ^4^Department of Neurosurgery, The Second Affiliated Hospital, School of Medicine, Zhejiang University, Hangzhou, China; ^5^Clinical Research Center for Neurological Diseases of Zhejiang Province, Hangzhou, China; ^6^MOE Frontier Science Center for Brain Science and Brain-Machine Integration, School of Brain Science and Brain Medicine, Zhejiang University, Hangzhou, China

**Keywords:** aging, glymphatic system, time-of-day, diffusion MRI, influx, efflux

## Abstract

Advanced age, accompanied by impaired glymphatic function, is a key risk factor for many neurodegenerative diseases. To study age-related differences in the human glymphatic system, we measured the influx and efflux activities of the glymphatic system via two non-invasive diffusion magnetic resonance imaging (MRI) methods, ultra-long echo time and low-b diffusion tensor imaging (DTI*_*low–b*_*) measuring the subarachnoid space (SAS) flow along the middle cerebral artery and DTI analysis along the perivascular space (DTI-ALPS) along medullary veins in 22 healthy volunteers (aged 21–75 years). We first evaluated the circadian rhythm dependence of the glymphatic activity by repeating the MRI measurements at five time points from 8:00 to 23:00 and found no time-of-day dependence in the awake state under the current sensitivity of MRI measurements. Further test–retest analysis demonstrated high repeatability of both diffusion MRI measurements, suggesting their reliability. Additionally, the influx rate of the glymphatic system was significantly higher in participants aged >45 years than in participants aged 21–38, while the efflux rate was significantly lower in those aged >45 years. The mismatched influx and efflux activities in the glymphatic system might be due to age-related changes in arterial pulsation and aquaporin-4 polarization.

## Introduction

Advanced age is a key risk factor for many neurodegenerative diseases such as Alzheimer’s disease (Association, 2018; Hou et al., 2019), Parkinson’s disease (PD) (Poewe et al., 2017), and Huntington’s disease (Diguet et al., 2009). A common feature of these diseases is age-associated accumulation of protein aggregates, such as hyperphosphorylated tau and amyloid-β in Alzheimer’s disease (Therneau et al., 2021). The glymphatic system is a newly discovered and defined perivascular pathway that facilitates recirculation of cerebrospinal fluid (CSF) through the brain parenchyma and supports clearance of interstitial solutes, including tau and amyloid-β (Boespflug and Iliff, 2018). Impaired glymphatic function has been reported in many neurodegenerative diseases, including Alzheimer’s disease (Harrison et al., 2020) and PD (Shen et al., 2022). Thus, characterizing the glymphatic function in aging and neurodegenerative diseases may provide new biomarkers for early diagnosis of these diseases and may enable more efficient treatment (Buccellato et al., 2022).

In rodents, the glymphatic system is impaired during aging (Benveniste et al., 2019). The glymphatic system includes three essential physiological activities: (1) CSF influx from the subarachnoid space into the periarterial space (called “influx”), (2) aquaporin-4 (AQP4)-dependent exchange between periarterial CSF and the parenchymal interstitial fluid (ISF) (called “exchange”), and (3) perivenous efflux of brain interstitial waste products (called “efflux” or “clearance”) (Iliff et al., 2013b). The perivenous conduits are connected to the lymphatic circulation outside the brain via lymphatic vessels in the dura or cranial nerves (Zhou et al., 2020). Using fluorescent CSF tracers and optical imaging methods, researchers have demonstrated a dramatic decrease in glymphatic influx activity in older mice/rats compared with that of younger mice/rats (Kress et al., 2014; Giannetto et al., 2020). An immunofluorescence study revealed a loss of perivascular AQP4 polarization and reduced CSF-ISF exchange activity in aging mice (Kress et al., 2014); thus, the glymphatic efflux activity slowed with age. This was demonstrated by injecting intraparenchymal radiotracers in mice (Kress et al., 2014) and intracisternally administering MRI contrast agent along with dynamic contrast-enhanced (DCE) MRI acquisition and kinetic analysis in rats (Li et al., 2022). Because lymphatic vessels are the major outflow pathways of the glymphatic system, the significant decreases in CSF outflow in lymphatic vessels with age further supports glymphatic function impairment (Ma et al., 2017; Da Mesquita et al., 2018).

Despite advances in animal research, evidence of the glymphatic system in aging human brains remains scarce, and non-invasive imaging methods for visualizing and quantifying the human glymphatic system are limited. Previously, we administered intrathecal MRI contrast agent as a CSF tracer and performed T1-weighted MRI before and at multiple time points after tracer administration and observed decreased clearance of both the glymphatic pathway and putative meningeal lymphatic vessels during aging in human brains (Iliff et al., 2013b; Zhou et al., 2020). However, the patient composition in this observational cohort study was complex and included patients with encephalitis, peripheral neuropathy, and possible cerebral amyloid angiopathy (Iliff et al., 2013b). Studying patients without neurodegenerative pathologies is still needed, along with non-invasive imaging methods. Additionally, other physiological activities of glymphatic function, including influx and exchange activity, have not been described in aging human brains.

Taoka et al. (2017) proposed an index for diffusion tensor imaging (DTI) analysis along the perivascular (specifically perivenous) space (DTI-ALPS) to indicate the glymphatic efflux function. DTI-ALPS measures the flow-induced pseudo-diffusivity of perivascular space water along the medullary veins, which run perpendicular to the wall of the lateral ventricle body. This was further normalized by the apparent diffusivities measured perpendicular to both the medullary veins and the fibers in the region of the projection fibers and association fibers (superior longitudinal fascicles) to eliminate potential changes due to water self-diffusivity (Taoka et al., 2017). This method has revealed decreased glymphatic function in many brain disorders, including Alzheimer’s disease (Kamagata et al., 2022), PD (Shen et al., 2022), and multiple sclerosis (Carotenuto et al., 2022). We compared DTI-ALPS with DCE-MRI results after intrathecal administration of MRI contrast agent and found a good correlation between these two glymphatic efflux indexes (Zhang et al., 2021). We also found that the DTI-ALPS-index was weakly correlated with age in patients aged 64 ± 9 years with cerebral small vessel diseases (*r* = −0.163) (Zhang et al., 2021), indicating that glymphatic efflux activity might be impaired with aging in human brains.

Regarding glymphatic influx activity, Harrison et al. (2018) proposed a novel diffusion-weighted MRI method with ultra-long echo time (TE), a low *b*-value, and multiple directions (DTI*_*low–b*_*) to measure CSF influx movements in the subarachnoid space surrounding the middle cerebral artery (MCA) in rats. The ultra-long TE was used to suppress non-CSF MRI signals because CSF has a much longer T2 than do vascular water and other tissue components. The low *b*-value and multiple directions in DWI enable detecting pseudo-diffusivity (i.e., higher apparent diffusion coefficients) induced by pseudorandom flows of the CSF in the MCA SAS (Bito et al., 2021). The SAS surrounding the MCA is one of the primary periarterial influx routes of the CSF into the parenchyma (i.e., glymphatic influx pathway), which is well characterized in several rodent studies using fluorescence CSF tracers or MRI contrast agent (Iliff et al., 2013b; Kress et al., 2014; Bedussi et al., 2017; Pizzo et al., 2018). Additionally, Iliff et al. (2013a) demonstrated that the CSF flow inside the MCA SAS measured by DTI*_*low–b*_* is driven by cerebral arterial pulsation, which is consistent with previous studies using fluorescence imaging or DCE-MRI (Iliff et al., 2013a; Kress et al., 2014) and demonstrates the method’s reliability. However, implementation of DTI*_*low–b*_* in human studies is limited (Bito et al., 2021), and no application has been found in human aging studies.

Several rodent studies have reported that glymphatic activity is strictly controlled by circadian rhythms (Hablitz et al., 2020). In humans, studies have reported that the total CSF volume (Trefler et al., 2016) and perivascular space (PVS) volume in white matter showed time-of-day (TOD) dependence (Barisano et al., 2021). However, whether the perivascular fluid flow shows TOD dependence in humans remains unknown. Such information is important to properly implement and interpret diffusion MRI measurements of glymphatic activity in human brains, as most studies have not reported the TOD of the acquisition.

**FIGURE 1:**
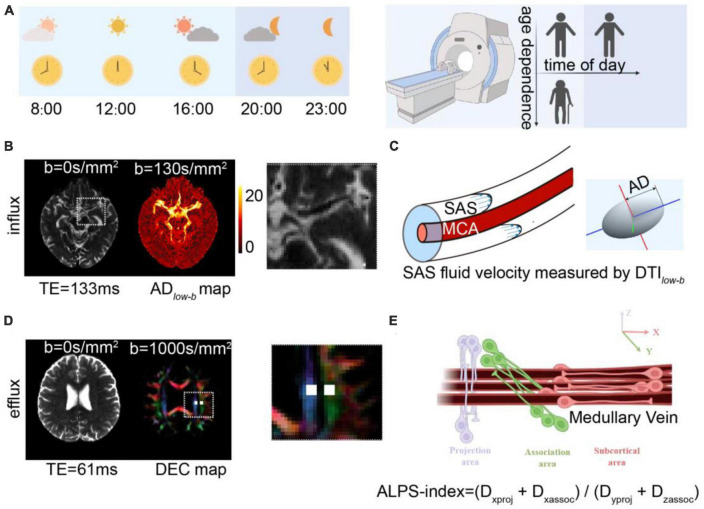
Diffusion MRI for measuring glymphatic influx and efflux activity. **(A)** Illustration of the experimental design, where 22 participates aged 21–75 years underwent MRI scanning to investigate age dependence of the glymphatic system and 10 of the 22 participates (aged 24 ± 4 years) underwent five MRI examinations at five time points to measure the circadian rhythm of the human glymphatic system. **(B)** Glymphatic influx activity was measured via DTI*_*low–b*_* with ultra-long TE and a low *b*-value on the subarachnoid space (SAS) of the middle cerebral artery (MCA) M1 segment. Representative DTI*_*low–b*_* b0 and axial diffusivity (AD*_*low–b*_*) maps are shown. The MCA SAS is enlarged in the dashed square. **(C)** MCA SASfluid flow, with the flow speed evaluated with the axial diffusivity (AD) of the diffusion tensor aligning parallel to the physiological structure of the MCA SAS. **(D,E)** Glymphatic efflux activity was evaluated via DTI-ALPS on the PVS of the medullary veins. Representative DTI-ALPS b0 map and direction-encoded color (DEC) of the uppermost layer of the lateral ventricle body are shown, with the projection area (blue) and association fibers (green). The unilateral ALPS-index was defined as [(D_xproj_ + D_xassoc_)/(D_yproj_ + D_zassoc_)], The average value of the bilateral ALPS-index was calculated as the glymphatic efflux activity index, where D_xproj_, D_xassoc_, D_yproj_, and D_zassoc_ are the diffusivities in the x direction of the projection fiber area, x direction of the association fiber area, y direction of the projection fiber area, and z direction of the association fiber area.

Here, we (1) explored the age-dependence glymphatic influx and efflux activity in human brains on the same participants over a large age range using non-invasive diffusion MRI methods and (2) determined whether these glymphatic metrics showed TOD dependence. We recruited 22 healthy participants aged 21–75 years. DTI*_*low–b*_* was implemented to measure the CSF flow along the SAS surrounding the MCA in human brains, which is proposed to reflect the flow properties in one of the primary CSF influx routes into the brain parenchyma. Additionally, the DTI-ALPS was implemented to measure the global glymphatic efflux activity. Finally, ten participants (aged 24 ± 4 years) underwent five DTI*_*low–b*_*, DTI-ALPS, and other MRI scans in a single day (from 8:00 to 23:00) to study TOD dependence (Figure 1A).

## Materials and methods

### Participants

We consecutively recruited healthy volunteers from our local community. All participants provided written informed consent before study commencement and after receiving approval from the Ethics Committee of the Second Affiliated Hospital, School of Medicine, Zhejiang University. The exclusion criteria were (1) any MRI contraindications; (2) serious head injury (resulting in loss of consciousness) or receipt of intracranial surgery; (3) cancer; (4) abnormal brain MRI findings such as head trauma, hemorrhaging, non-lacunar infarction and other space-occupying lesions; (5) definitive peripheral neuropathy or spinal cord disease; and (6) dementia or stroke. Twenty-two participants were finally enrolled.

### MRI scanning

Each participant was examined with a 3.0 T MRI clinical scanner (MAGNETOM Prisma, Siemens Healthcare, Erlangen, Germany), using a Nova 64-channel head radio frequency (RF) coil. MRI scans included three-dimensional (3D) magnetization prepared with two rapid gradient echoes (MP2RAGE) T1-weighted images, DTI-ALPS and DTI*_*low–b*_*. Total scan time was approximately 20 min per participant. The MP2RAGE images were acquired with 1.0 mm × 1.0 mm × 1.2 mm resolution, echo time (TE)/repetition time (TR): 2.9/5000 ms, flip angles: 4 and 5°, inversion times: 700 and 2500 ms, and generalized autocalibrating partially parallel acquisitions (GRAPPA): 3. Glymphatic influx activity was measured via DTI*_*low–b*_*, which was acquired with *b* = 130 s/mm^2^ in 30 directions (single repetition at each direction) and *b* = 0 s/mm^2^ with a single repetition, 1.0 mm × 1.0 mm × 3.0 mm resolution, 20 slices, no slice gap, GRAPPA: 2, TE/TR: 133/4000 ms, and Δ/δ = 64.9/34 ms. Glymphatic efflux activity was measured via DTI-ALPS with *b* = 1000 s/mm^2^ in 30 directions (single repetition at each direction) and *b* = 0 s/mm^2^ with a single repetition, 2.0 mm × 2.0 mm × 2.0 mm resolution, 20 slices, no slice gap, GRAPPA: 2, TE/TR: 61/4000 ms, and Δ/δ = 29.9/14.9 ms.

For the TOD experiments (Figure 1A), 10 participates (aged 24 ± 4 years, five men, five women) underwent MRI scans at five time points (8:00, 12:00, 16:00, 20:00 and 23:00) in 1 day. During scanning, volunteers were asked to remain awake and could react to a calling system equipped on the MRI scanner.

### MRI data pre-processing

Artifacts due to eddy currents and motion were corrected with the DIFFPREP (Irfanoglu et al., 2015) and EPI geometric distortion corrections along with the DWIs of the opposite phase encoding direction using DR_BUDDI (Irfanoglu et al., 2015) in TORTOISE (Pierpaoli et al., 2010). The preprocessed DWIs from each participant were then fitted to the DTI model (Basser et al., 1994) to generate DTI metrics. For voxel-wise analysis, the DTI metrics, including axial diffusivity (AD), fractional anisotropy (FA), mean diffusivity (MD), and direction-encoded color (DEC) maps were generated using TORTOISE. For the regions of interest (ROI)-wise analysis, the DTI metrics, including AD and FA, were generated using in-house programs developed in MATLAB (The Math-Works, Natick, MA, USA).

### DTI_*low–b*_ on the SAS of the MCA

The glymphatic system influx was measured as the AD of the MCA SAS through DTI*_*low–b*_* (Figures 1B, C). In the DTI*_*low–b*_* analysis, the ROIs in the SAS at the M1 stage of the left and right MCA (MCA SAS) were carefully drawn by one neuroradiologist with 5-years of experience (JH) and further checked by a senior neuroradiologist with 9-years of experience (YZ), both of whom were blind to the group information, on the distortion-corrected DTI*_*low–b*_* b0 image using in-house programs. The DTI*_*low–b*_* voxels in each ROI in each hemisphere were averaged to construct the 3 × 3 diffusion tensor < D_3 × 3_ > for this ROI. Subsequently, eigenvalues and eigenvectors were calculated after diagonalizing the diffusion tensor matrix < D_3 × 3_ >. Given the three eigenvalues (λ_1_, λ_2_, and λ_3_, sorted from maximum to minimum) and corresponding eigenvector, the ellipsoid was displayed in the 3D coordinate system, where the maximum eigenvalue, λ_1_, was defined as the AD*_*low–b*_* (Figure 1C). The fractional anisotropy of DTI*_*low–b*_* (FA*_*low–b*_*), which reflects the flow anisotropy rather than diffusion anisotropy (FA), was derived by calculating the three eigenvalues of DTI*_*low–b*_*, similar to FA in conventional DTI (Basser et al., 1994). Finally, the AD*_*low–b*_* values of the bilateral MCA SAS ROIs were averaged and the mean AD*_*low–b*_* was further taken for TOD-dependence, test–retest, and age-dependence analysis.

### ALPS-index calculation

The glymphatic system efflux was measured as the ALPS-index through DTI-ALPS (Figures 1D, E). To calculate the DTI-ALPS index, we used a previous described protocol in the (Zhang et al., 2021), in which the DTI-ALPS index was calculated without SWI and was significantly correlation to the glymphatic efflux activity measured with DCE-MRI with intrathecal administration of MRI contrast agent. The medullary veins run perpendicular to the wall of the lateral ventricle body at the uppermost layer of the lateral ventricle body (Okudera et al., 1999), allowing measurement of the diffusivity without visualizing the veins. In the DTI-ALPS analysis, the ROIs of the projection fibers and association fiber placement were processed through registration then carefully checked and manually adjusted by the investigators. Each ROI was defined as a 4 mm × 4 mm rectangular area as per the Montreal Neurological Institute (MNI) template. MNI coordinates of the centers of the left and right ROIs were (24, −12, 24) and (−28, −12, 24) in the projection fibers and (36, −12, 24) and (−40, −12, 24) in the association fibers. For more precise registration, an MNI FA template (JHU-ICBM-FA-2 mm)^1^ was used for each participant’s FA images. Registration from the MNI space to the individual space was achieved via rigid registration followed by non-linear registration using Advanced Normalization Tools (ANTs).^2^ All ROIs for each individual were visually inspected and two experienced neurologists (JH and YZ) who were blinded to the experimental data, made minor manual corrections if necessary to confirm the accuracy of the location by ensuring that only blue voxels were included in the ROIs on the projected fibers and only green voxels were included in the ROIs on the associated fibers. The ALPS-index is calculated as the ratio of two sets of diffusivity values perpendicular to the dominant fibers in the tissue (Figure 1E), i.e., the ratio of the average *x*-axis diffusivity in the area of the projection fibers (D_*x*_*_*proj*_*) and the *x*-axis diffusivity in the area of association fibers (D_*x*_*_*assoc*_*) to the average *y*-axis diffusivity in the area of projection fibers (D_*y*_*_*proj*_*) and the *z*-axis diffusivity in the area of association fibers (D_*z*_*_*accoc*_*) as [(D_*x*_*_*proj*_* + D_*x*_*_*assoc*_*) / (D_*y*_*_*proj*_* + D_*z*_*_*assoc*_*)]. Finally, the ALPS-indexes of each hemisphere were averaged and used for further analysis.

### Circadian rhythm analysis

Circadian rhythms were evaluated via one-factor (TOD) analysis of variance (ANOVA) and cosinor analysis (Nelson et al., 1979; Minors and Waterhouse, 1988). The ANOVA enabled determining whether the variance between time points was significantly greater than the random variation within them. The cosinor analysis enabled determining whether the data are better described by a cosine curve than by a straight line. A “significant” fit (*p* < 0.05) is a fit where the chance that the data are as well fitted by a horizontal line as by a cosine curve is <5% (Minors and Waterhouse, 1988).

### Test–retest measurement reliability

The test–retest repeatability of the scanning at different time points was evaluated using intraclass correlation coefficients (ICCs) and Bland-Altman analysis to determine the coefficients of variation (CVs), mean differences, and repeatability coefficients. The ICC ranges considered to have excellent, good, moderate, and poor repeatability were ≥0.90, 0.75–0.89, 0.50-0.74, and <0.50, respectively (Koo and Li, 2016). CVs for interscan (test–retest) reproducibility were calculated based on the variances, using the variance due to within-participant variance between scan 1 and 2 (σ_*w*_^2^) and variances due to random noise (σ_*e*_^2^) along the concentration mean μ (Gasparovic et al., 2011).


$$C\,V=\frac{\sqrt{\sigma_{\text{w}}^{2}+\sigma_{\text{e}}^{2}}}{\mu }$$

### Statistical analyses

Before performing the statistical analyses, we screened for outliers using the Grubbs’ test (i.e., the Extreme Studentized Deviate method) with a significance level of α = 0.01.^3^ For the group-level analysis, the younger group included 11 participants aged 21–38 years (mean: 25.1 years), and the older group included 11 participants aged 48–75 years (mean: 62.6 years). The AD*_*low–b*_* and ALPS-index across the younger and older groups are shown as the mean ± standard deviation and compared using unpaired Student’s *t*-tests. The difference between the MCA SAS and quadrigeminal cistern was calculated using paired Student’s *t*-tests. The widths of the left and right MCA SAS across the younger and older groups are shown as the mean ± standard deviation in the Supplementary Information and compared via ANOVA. Spearman’s correlation tests were used to analyze the age dependence of and the correlation between the AD*_*low–b*_* and ALPS-index. The circadian rhythm was evaluated using ANOVA and cosinor analysis. Except for the cosinor analysis, all statistical analyses were performed in GraphPad Prism 8. The cosinor analysis was performed using in-house programs developed in MATLAB. *P* < 0.05 was considered statistically significant.

## Results

### Participant demographics

We recruited 22 participants in total and divided them into two groups: the younger group (aged <45 years) and the older group (aged >45 years; Table 1).

**TABLE 1:** Participants’ demographic information.

Group	Young	Old
Number of participants	11	11
Women	54.5%	27.3%
Age range (years), (average)	21–38 (25.1)	47–75 (62.6)

### SAS fluid of the MCA M1 showed anisotropic diffusion in DTI_*low–b*_

**FIGURE 2:**
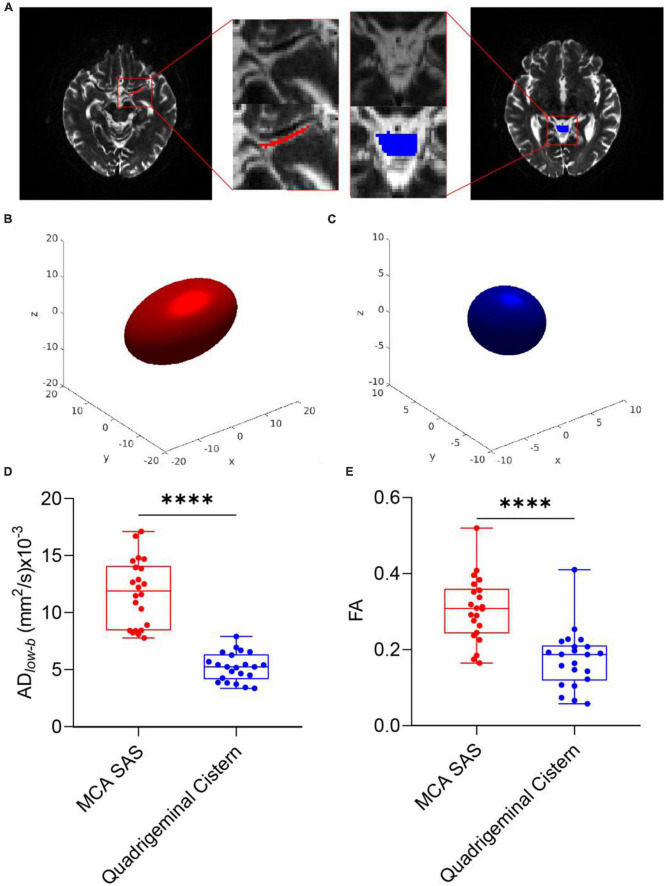
DTI*_*low–b*_* detected larger apparent diffusivity in the SAS of the MCA. **(A)** Representative ROIs of the SAS at the MCA M1 stage (red) and quadrigeminal cistern (blue) in one participant, overlaid on the b0 images of DTI*_*low–b*_*. **(B,C)** Ellipsoid-shaped tensor reconstructed from the two ROIs in panel **(A)** is displayed on the *x*-*y*-*z* axis. **(D,E)** Boxplot of FA and AD*_*low–b*_* values of the MCA SAS and quadrigeminal cistern for all participates (*n* = 22). Statistical analysis was performed using paired Student’s *t*-tests, with ****p < 0.0001.

Figures 2A–C shows representative b0 images of DTI*_*low–b*_*, the ROI of the SAS in MCA stage M1 and the quadrigeminal cistern and the reconstructed ellipsoid-shaped diffusion tensors. The quadrigeminal cistern was selected as a control region with no flow or much slower flow than those of the MCA SAS. Expectedly, the diffusion tensor of the quadrigeminal cistern water showed relatively low FA*_*low–b*_* and no clear direction preference. The diffusion tensor of the SAS fluid in MCA stage M1 showed a clear anisotropic property with larger apparent diffusivity which is consistent with the findings of Sepehrband et al. (2019), who also found the diffusion of the non-parenchymal fluid within SAS was anisotropic. Both the FA and AD*_*low–b*_* of DTI*_*low–b*_* were significantly higher in the MCA SAS than in the quadrigeminal cistern (*p* < 0.001; Figures 2D, E), demonstrating the pseudo-diffusivity induced by the SAS fluid reflected anisotropic diffusivity.

### Glymphatic influx and efflux activity detected by diffusion MRI showed no TOD dependence

**FIGURE 3:**
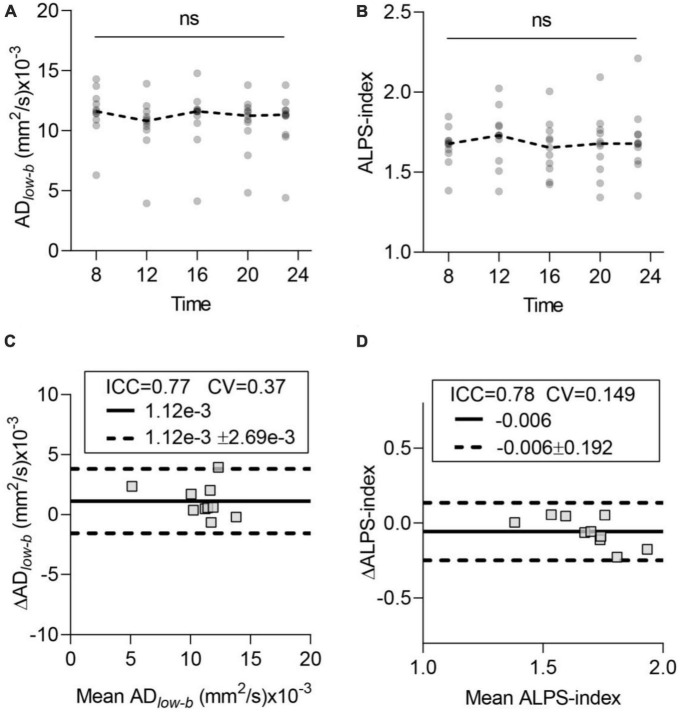
Time-of-day (TOD) test and test–retest repeatability of the glymphatic system measurements. **(A,B)** Neither AD*_*low–b*_* of the MCA SAS fluid nor the ALPS-index differed significantly in the data acquired at the five time points. Each participant’s data are shown as gray dots, and group-averaged results are shown as dashed lines. **(C,D)** Bland-Altman plots of the test–retest results (8:00 and 12:00) of AD*_*low–b*_* of the MCA SAS and ALPS-index. Each participant’s data are shown as gray boxes. The ICC, CV, MD (solid dark line), and MD ± repeatability coefficient (dashed black lines) are shown. CV, coefficient of variation; ICC, intraclass correlation coefficient; MD, mean diffusivity. *n* = 10, aged 24 ± 4 years. ns, non-significant by ANOVA.

Figures 3A, B show the time course of the glymphatic system influx and efflux measured by DTI*_*low–b*_* and DTI-ALPS, respectively. The acquired data did not significantly differ among the five time points in either the AD*_*low–b*_* or ALPS-index by ANOVA (*p* > 0.05). Cosinor analysis also confirmed that neither the AD*_*low–b*_* nor the ALPS-index differed significantly from the straight lines (*p* > 0.05). Thus, neither the AD*_*low–b*_* nor the ALPS-index had TOD dependence from 8:00 to 23:00 for the participants while awake.

### Test–retest repeatability of the AD_*low–b*_ and ALPS-index

To test the repeatability of the influx and efflux measured via diffusion MRI, we selected two datasets (acquired at 8:00 and 12:00) for scan-rescan analysis. Bland-Altman plots showed the test–retest repeatability of the average measurements of AD*_*low–b*_* in the MCA SAS (Figure 3C) and ALPS-index (Figure 3D). AD*_*low–b*_* showed good repeatability (ICC = 0.77), and the ALPS-index showed good repeatability (ICC = 0.78), demonstrating the high repeatability and reliability of both the AD*_*low–b*_* and ALPS-index.

### Glymphatic influx activity measured via DTI_*low–b*_ increased with aging

**FIGURE 4:**
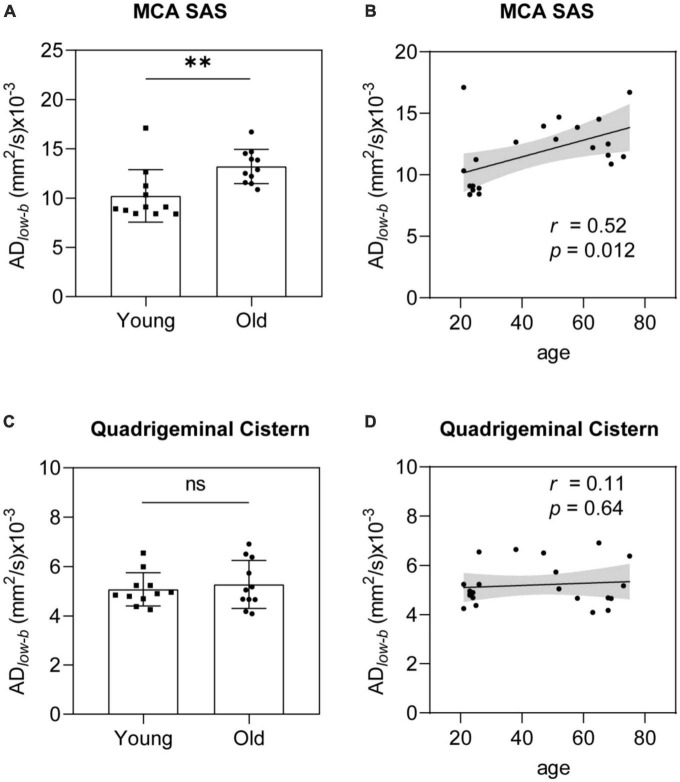
AD*_*low–b*_* of the MCA SAS increased significantly with age. **(A,B)** AD*_*low–b*_* values of the MCA SAS were significantly higher in the older group (*n* = 11) than in the younger group [*n* = 11, **(A)**] in the group analysis and were significantly positively correlated with age in the linear regression analysis **(B)**. **(C,D)** For the control region (i.e., the quadrigeminal cistern), the AD*_*low–b*_* did not differ significantly in the group analysis **(C)** or with age **(D)**. Statistical analysis was performed using Student’s *t*-tests, with ns as non-significant (*p* ≥ 0.05), ^**^p < 0.01.

Group analysis was used to verify differences between the older and younger participants. The AD*_*low–b*_* values of the MCA SAS fluid were significantly higher in the older group than in the younger group (*p* = 0.0054; Figure 4A). The AD*_*low–b*_* of the quadrigeminal cistern did not significantly differ between the groups (*p* = 0.58, Figure 4C). Linear regression analysis showed a significant positive correlation between AD*_*low–b*_* values and age in the MCA SAS region (*p* = 0.012, *r* = 0.52; Figure 4B) but not in the quadrigeminal cistern (*p* = 0.64, *r* = 0.11; Figure 4D). These results demonstrate that the AD*_*low–b*_* of the SAS fluid along the MCA increased with age, suggesting that the fluid flow along the MCA SAS became faster with age.

### Glymphatic efflux activity measured by DTI-ALPS decreased with age

**FIGURE 5:**
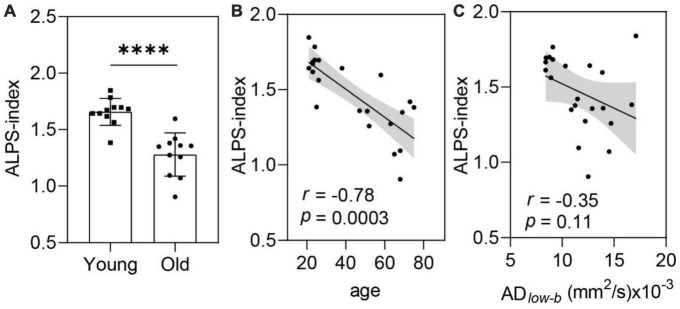
The ALPS-index was negatively correlated with age. **(A,B)** ALPS-index values were significantly lower in the older group (*n* = 11) than in the younger group [*n* = 11, **(A)**] in the group analysis and significantly negatively correlated with age in the linear regression analysis **(B)**. **(C)** The ALPS-index was negatively correlated (near significance) with the AD*_*low–b*_* of the MCA SAS. Group analysis was performed using Student’s *t*-test. ns: non-significant (*p* ≥ 0.05), ****p < 0.0001.

Along the perivascular space-index values were significantly smaller in the older group than in the younger group (*p* < 0.001; Figure 5A). Linear correlation analysis revealed a significant negative correlation between ALPS-index and age (*p* = 0.0003, *r* = −0.78; Figure 5B). These results suggest that glymphatic efflux activity measured by the ALPS-index decreased with age. Comparing the ALPS-index and AD*_*low–b*_* of the MCA SAS revealed a weak negative, but not significant, correlation (*r* = −0.35, *p* = 0.11; Figure 5C).

## Discussion

Knowledge of age-dependent glymphatic activity in the human brain is important for understanding aging. Here, we implemented and evaluated two diffusion MRI methods (DTI*_*low–b*_* and DTI-ALPS*)* to measure glymphatic influx and efflux activity in the same participants over a large age range. DTI*_*low–b*_* enabled successfully detecting a larger apparent diffusion coefficient (ADC) and diffusion anisotropy induced by the CSF flow in the MCA SAS. Glymphatic function derived from both DTI*_*low–b*_* and DTI-ALPS showed no TOD dependence from 8:00 to 23:00, suggesting that these metrics are not TOD-dependent, and further implementation of these measurements will not account for TOD. Finally, the AD*_*low–b*_* increased, and the ALPS-index decreased with age.

The lack of TOD dependence for both DTI*_*low–b*_* and DTI-ALPS in awake participants between 8:00 and 23:00 and the low variance of the AD*_*low–b*_* and ALPS-index throughout the day suggests that both diffusion methods can provide reproducible results and that the TOD effect can be ignored in the studied time range as long as patients remain awake. In anesthetized mice, the glymphatic influx activity, clearance efficiency and perivascular AQP4 polarization all show circadian rhythms, and their changes can be well described by a 24-h cosine function (Hablitz et al., 2020). Our results were unsurprising, as previous studies also demonstrated that glymphatic activity occurs mainly during sleep, whereas the awake brain has limited glymphatic activity (Xie et al., 2013; Hablitz et al., 2020). Hablitz et al. (2020) conducted the same study on awake mice and also found no circadian control of glymphatic influx. The limited sensitivity of the MRI measurements used in this study might also have induced this lack of TOD dependence.

We found that glymphatic efflux activity assessed via DTI-ALPS decreased with age over in a large age range of 21–75 years, which is consistent with our previous study on cerebral small vessel disease (CSVD) patients with a narrower age range (>60 years) using the same DTI-ALPS method. Decreased glymphatic efflux activity was also observed in patients undergoing lumbar puncture, who were administered intrathecal MRI contrast agent as a CSF tracer for T1-weighted MRI before and at multiple time points after tracer administration (Zhou et al., 2020). This is consistent with the CSF production rate, which decreases with age in both humans (May et al., 1990) and rodents (Chiu et al., 2012; Liu et al., 2020).

As the CSF runs along the SAS, evaluation of the AD*_*low–b*_* of the SAS fluid around the straight portion of the MCA segment could reflect the CSF influx activity of the glymphatic pathway. This was demonstrated in a study on rats, which revealed a 300% increase in apparent diffusivity of the SAS CSF along the vessel direction as the vessels pulsated with each heartbeat (Harrison et al., 2018). No such cardiac-cycle dependence was observed on the apparent diffusivity measured perpendicular to the vessel directions, suggesting the advantages of AD*_*low–b*_* in measuring CSF flow along vessel directions. This is also consistent with other glymphatic influx studies using fluorescence tracers or MRI contrast agents (Iliff et al., 2013a; Mestre et al., 2018). A recent particle-tracking velocimetry study on live mice revealed the CSF flow in the periarterial space following pipe Poiseuille flow with zero velocity at the PVS walls (Mestre et al., 2018). The effect of the velocity shear induced pseudo-diffusion with larger ADC along the flow direction. The analytical formula for the relationship between ADC (i.e., AD*_*low–b*_*) and flow velocity in the case of SAS pipe Poiseuille flow (Thomas, 2019) is as follows:


$${\text{AD}}_{low-b}={\text{D}}_{\text{CSF}}+{\text{a}}^{2}{\text{u}}^{2}{}_{\max}/192{\text{D}}_{\text{CSF}},$$

where D_*CSF*_ is the self-diffusion coefficient of CSF, “a” is the inner radius of the pipe, and u_*max*_ is the maximum flow velocity of the pipe (i.e., flow velocity in the pipe center). Using *a* = 1.5 mm (Supplementary Figure 1), D_*CSF*_ = 4 mm × 10^–3^ mm/s as the mean diffusivity in the quadrigeminal cistern, and AD*_*low–b*_* for the younger group defined as 10 mm × 10^–3^ mm/s, the expected u_*max*_ is 45.3 μm/s, which is in the same order of the CSF flow velocity measured in the PVS of mice (10–40 μm/s) (Kelley, 2021). Because the D_*CSF*_ (Figures 4C, D) and MCA SAS width showed no age dependence (Supplementary Figure 1), the increased AD*_*low–b*_* suggests that the CSF influx flow velocity inside the MCA SAS increased with age.

Several factors involved with aging can induce increased flow velocity along the periarterial SAS, possibly because the cerebral artery pulsatility and stiffness increase with age owing to arteriosclerosis and other vessel degeneration (Iliff et al., 2013c; Tarumi et al., 2014; Mitchell and Powell, 2020; Fico et al., 2022). Iliff et al. found that perivascular CSF influx was mainly driven by cerebral arterial pulsation, and increased arterial pulsatility after administering the adrenergic agonist, dobutamine, significantly increased the perivascular CSF influx. Additionally, decreased arterial pulsatility after unilateral ligation of the internal carotid artery significantly decreased the perivascular CSF influx (Iliff et al., 2013c). Decreased blood flow velocities and concomitantly increased pulsatility occur in the middle, anterior and posterior cerebral arteries with advanced age, especially in those aged >40 years (Ackerstaff et al., 1990; Krejza et al., 1999; Xu et al., 2017). Further, a rodent study showed that glymphatic system influx increased with the pulsatility of vascular increase (Mestre et al., 2018; Cao et al., 2022), possibly owing to decreased intracranial pressure. Another study reported that the decrease in intracranial pressure after acute ischemic stroke could directly lead to rapid CSF influx (Xu et al., 2017). Studies have found decreased cerebral blood flow and brain atrophy in aging brains (Krejza et al., 1999; Cao et al., 2022). Both the decreased cerebral blood flow and brain atrophy can lead to decreased intracranial pressure (Krejza et al., 1999; Omileke et al., 2021), which has also been shown to decrease with age (Gur et al., 1991).

In our dataset, we observed a negative correlation between DTI-ALPS and AD*_*low–b*_* (Figure 5C), although this association was not statistically significant. This mismatch between glymphatic influx and efflux activity with age is interesting. Such a mismatch was also observed in AQP4-knockout mice, in which the glymphatic efflux or clearance activity was largely suppressed, but movement of the perivascular tracer along the periarterial spaces was not significantly altered (Iliff et al., 2013b). The influx of CSF along the periarterial space (e.g., MCA SAS) is considered to be the initial driving force behind the glymphatic clearance system (Jessen et al., 2015; Wostyn et al., 2021). But, the perivascular AQP4 is the key membrane channel gating the exchange between periarterial CSF and the interstitial ISF (Iliff et al., 2013b). With perivascular AQP4 dysfunction, the unaltered or increased periarterial CSF flow can still result in decreased glymphatic clearance, as periarterial CSF cannot flow efficiently into the interstitial space. The association between AQP4 downregulation and the glymphatic efflux/clearance activity suppression has been demonstrated in many studies (Pedersen et al., 2018). Thus, the altered perivascular AQP4 function/expression along aging could be a potential factor contributing to this mismatch between periarterial and perivenular CSF motion observed in this study. Indeed, Kress et al. (2014) reported that AQP4 polarization on the astroglial end-feet processes surrounding the cortical penetrating arterioles (but not the capillaries) was significantly reduced in 18-month-old mice compared with that of 2-to 3-month-old mice. However, these data were not corroborated in a recent study reporting that AQP4 expression in membranes next to the capillary endothelial cells and arterioles was independent of age in human frontal cortex (Zeppenfeld et al., 2017). Clearly, more studies are still needed to further explore the perivascular AQP4 expression in aging in more brian regions as both AQP4 expression and glymphatic activity show spatial heterogeneity across brain regions (Iliff et al., 2013a; Li et al., 2022). In addition, the permeability of AQP4 channel should also be investigated in future as it has been reported to be gated/regulated by metal ions, intracellular signaling pathways, antiepileptic drug, etc. (Yukutake and Yasui, 2010).

Several limitations and future works of this study should be clarified. The first limitation is the small sample size, though careful statistics were performed. It is highly desired to perform such study in a large sample. Second, DTI*_*low–b*_* and DTI-ALPS are presumed to measure glymphatic influx and efflux activities, respectively, mainly because of the anatomical locations of the two measurements (PVS or SAS surrounding arteries and veins, respectively). However, the flow direction of CSF in PVS was still under debate (Bakker et al., 2019), moreover, neither DTI*_*low–b*_* nor DTI-ALPS can show the CSF flow directions because they both measure the pseudo diffusivity induced by slow flow motion, which can be unidirectional or bidirectional or change direction over time. Third, both DTI*_*low–b*_* and DTI-ALPS were acquired without cardiac gating and presumed to be the average of the entire cardiac cycle. Glymphatic activity has been demonstrated to be largely driven by arterial pulsation (Mestre et al., 2018). For DTI*_*low–b*_*, Harrison et al. (2018) demonstrated that the AD*_*low–b*_* of the MCA SAS has strong cardiac-cycle dependence. Wen et al. (2022) found the cardiac-cycle dependence of whole-brain PVS ADC, although their DTI*_*low–b*_* protocol differed from that used in our study (their protocol included shorter TE and lower spatial resolution). However, the non-gated diffusivity measured via DTI*_*low–b*_* still showed sensitivity to manipulation of the glymphatic activity (injection of adrenoceptor agonist, dobutamine) (Harrison et al., 2018) or to aging (Wen et al., 2022), although further physiological validation is needed to test whether the CSF in SAS MCA was caused by the arterial pulse. Fourth, in this study, though a long-TE setup was implemented in DTI*_*low–b*_* to suppress non-CSF signal, there might still be non-CSF signal left. Another approach is to acquire DTI*_*low–b*_* at multiple *b*-values and fit the data with a bi-tensor model, in which one tensor is to describe CSF component considering perfusion effect and another tensor is to describe non-CSF tissue MR signal. Fifth, both DTI*_*low–b*_* and DTI-ALPS could can reflect only the glymphatic function on fixed and specific brain regions. Non-invasive MRI methods that can characterize the glymphatic function in the whole brain are needed. Finally, although we proposed that the altered perivascular AQP4 polarization along aging could be the potential factor contributing the mismatch between the glymphatic influx and efflux functions observed in this study, the direct evidence to demonstrate this hypothesis is still lacking and the development of non-invasive MRI method to measure the glymphatic exchange process *in vivo* is an important future research direction.

## Data availability statement

The raw data supporting the conclusions of this article will be made available by the authors, without undue reservation.

## Ethics statement

The studies involving human participants were reviewed and approved by the Ethics Committee of the Second Affiliated Hospital, School of Medicine, Zhejiang University. The patients/participants provided their written informed consent to participate in this study.

## Author contributions

All authors listed have made a substantial, direct, and intellectual contribution to the work, and approved it for publication.

## Footnotes

1
https://neurovault.org/images/1406/

2
https://stnava.github.io/ANTs/

3
https://www.graphpad.com/quickcalcs/grubbs1/
