# Supplementary material for: Age- and time-of-day dependence of glymphatic function in the human brain measured via two diffusion MRI methods
Source: Front Aging Neurosci. 2023 May 22;15:1173221. doi: 10.3389/fnagi.2023.1173221 (PMC10239807; doi:10.3389/fnagi.2023.1173221)
Supplement: Supplementary file 1 [file Image_1.pdf]

Supplementary Materials:

MCA SAS *width calculation*

The width of the SAS at the M1 stage of the left and right MCA (MCA SAS) were carefully counted by two neuroradiologists (J. H and B.L.), both of whom were blinded to the group information, on the distortion-corrected $DTI_{low\text{-}b}$ b0 image (the b0 image contrast is equivalent to that of T2 images) using in-house programs.

$DTI_{high\text{-}b}$ (b= 1000 s/mm^2) metrics of MCA SAS

The tensor metrics of conventional $DTI_{high\text{-}b}$ (b=1000 s/mm^2, used for DTI-ALPS) was also calculated using the same procedure as $DTI_{low\text{-}b}$. The ROIs of the MCA SAS defined in the $DTI_{low\text{-}b}$ spaces were registered to the $DTI_{high\text{-}b}$ space using a rigid transformation performed with FLIRT in FSL (http://www.fmrib.ox.ac.uk/fsl). The result shows that both AD and FA values of $DTI_{high\text{-}b}$ were significantly lower than those obtained from $DTI_{low\text{-}b}$ ($p < 0.0001$; S.I. Figure 2), respectively, indicating the flow in MCA SAS and PVS along medullary vein might not in the same scale as $DTI_{high\text{-}b}$ and $DTI_{low\text{-}b}$ are sensitive to slow motion and faster motion of molecules in between diffusion and convection regime, respectively.

Supplemental Figures

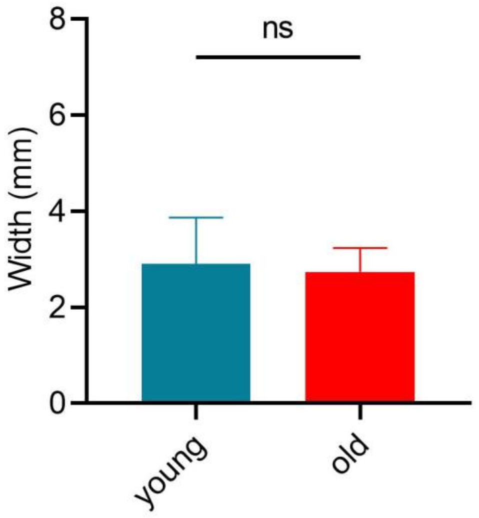

S.I. Figure 1. Width evaluation of the MCA SAS between groups. Comparison of the MCA SAS widths between the younger and older groups using unpaired Student's t-tests. ns: $p \geqslant 0.05$.

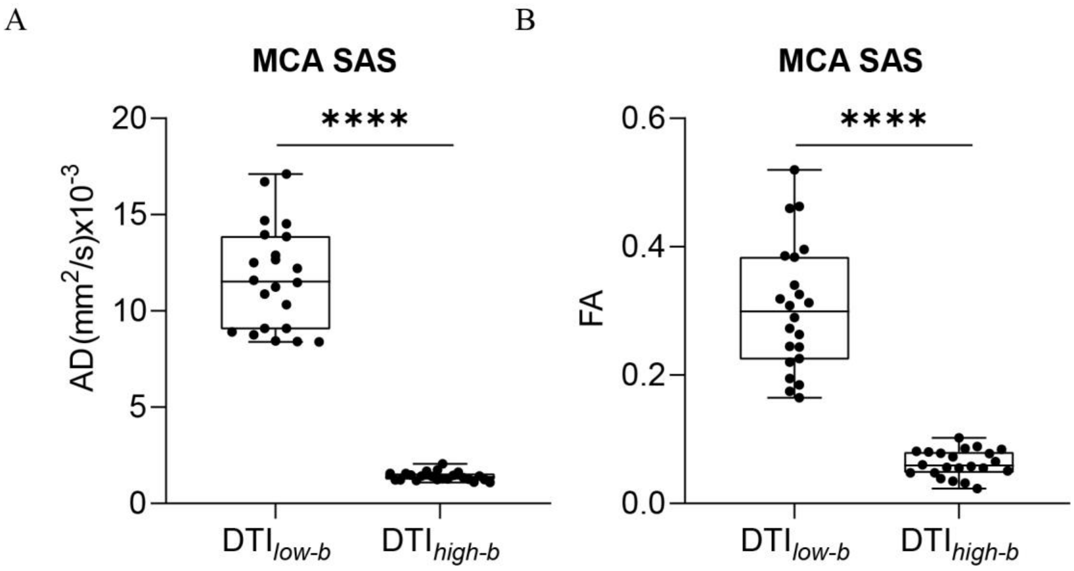

S.I. Figure 2. Comparison of the DTI*low-b* and DTI*high-b* in MCA SAS. Boxplot of AD **(A)** and FA **(B)** values of the DTI*low-b* and DTI*high-b* in MCA SAS for all participates (n=22). Statistical analysis was performed using paired Student's t-tests, with ****p<0.0001.
